# Effective contribution ratio of the molar during sequential distalization using clear aligners and micro-implant anchorage: a finite element study

**DOI:** 10.1186/s40510-023-00485-0

**Published:** 2023-10-09

**Authors:** Xulin Liu, Junjie Wu, Yuxun Cheng, Jie Gao, Yi Wen, Yubohan Zhang, Houzhuo Luo, Zuolin Jin, Yanning Ma

**Affiliations:** 1 State Key Laboratory of Military Stomatology & National Clinical Research Center for Oral Diseases & Shaanxi Clinical Research Center for Oral Diseases, Department of Orthodontics, School of Stomatology, Air Force Medical University, Xi’an, 710032 China; 2 Shanxi Medical University School and Hospital of Stomatology, Taiyuan, 030001 China

**Keywords:** Clear aligners, Finite element analysis, Microimplants, Molar distalization

## Abstract

**Introduction:**

This study aims to investigate the biomechanical effects of anchorage reinforcement using clear aligners (CAs) with microimplants during molar distalization. And also explores potential clinical strategies for enhancing anchorage in the sequential distalization process.

**Methods:**

Finite element models were established to simulate the CAs, microimplants, upper dentition, periodontal ligament (PDL), and alveolar bone. In group set I, the 2nd molars underwent a distal movement of 0.25 mm in group set II, the 1st molars were distalized by 0.25 mm after the 2nd molars had been placed to a target position. Each group set consisted of three models: Model A served as the control model, Model B simulated the use of microimplants attached to the aligner through precision cuts, and Model C simulated the use of microimplants attached by buttons. Models B and C were subjected to a series of traction forces. We analyzed the effective contribution ratios of molar distalization, PDL hydrostatic stress, and von Mises stress of alveolar bone.

**Results:**

The distalization of the 2nd molars accounted for a mere 52.86% of the 0.25-mm step distance without any reinforcement of anchorage. The remaining percentage was attributed to the mesial movement of anchorage teeth and other undesired movements. Models B and C exhibited an increased effective contribution ratio of molar distalization and a decreased loss of anchorage. However, there was a slight increase in the undesired movement of molar tipping and rotation. In group set II, the 2nd molar displayed a phenomenon of mesial relapse due to the reciprocal force produced by the 1st molar distalization. Moreover, the efficacy of molar distalization in terms of contribution ratio was found to be positively correlated with the magnitude of force applied. In cases where stronger anchorage reinforcement is required, precision cuts is the superior method.

**Conclusions:**

The utilization of microimplants in conjunction with CAs can facilitate the effective contribution ratio of molar distalization. However, it is important to note that complete elimination of anchorage loss is not achievable. To mitigate undesired movement, careful planning of anchorage preparation and overcorrection is recommended.

**Supplementary Information:**

The online version contains supplementary material available at 10.1186/s40510-023-00485-0.

## Introduction

Maxillary molar distalization is an effective non-extraction treatment option for class II malocclusions with mild crowding [1]. Traditional orthodontic procedures, such as the pendulum and distal jet, frequently result in unwanted tooth movement during distalization. These include distal tipping, molar extrusion, and incisor protrusion due to anchorage loss [2]. In recent years, many patients have sought clear aligner (CA) therapy (CAT) for aesthetic and comfort reasons. A significant development in CAT has been achieved with the growth of biomechanics and material science, which have improved therapeutic effectiveness. Simon et al. [3] reported that the overall mean efficacy of Invisalign® was 59%, and distalization of an upper molar was the most effective movement, with 87% efficacy when a distalization movement of 1.5–3.2 mm was prescribed. Ravera et al. [4] demonstrated that combining CAs with composite attachments and Class II elastics caused distalization of the maxillary first molars by 2.25 mm without remarkable crown tipping or vertical movements. Rossini et al. [5] reported that upper-molar distalization with clear aligners guarantees excellent control of the vertical dimension, representing an ideal solution for treating hyperdivergent or open bite subjects.

The setup in CA software can not accurately reflect the actual movement direction and distance of the teeth due to the limits of the materials and force application [3]. Supplementary devices are necessary when a more significant distalizing movement of more than 3 mm is performed to increase the predictability of orthodontic movement [6]. Because of the challenges associated with applying a couple of forces with a CA, the movement is mostly uncontrolled tipping with the center of rotation located between the center of resistance (CRes) and the apex of the tooth [7]. Furthermore, anchorage loss is unavoidable because molar distalization exerts a reciprocal stress on the anchorage teeth [8]. This may represent a substantial cause of alveolar defects, such as dehiscence and fenestration, in some patients with thin cortical plates in the anterior region [9]. Therefore, anchorage reinforcement is necessary during molar distalization using CAT [10].

Microimplant anchorage (MIA) can be used as an efficient skeletal anchorage for molar distalization to prevent anchorage loss with less treatment time. MIA has become a commonly used temporary anchorage device (TAD) [10]. The biomechanical versatility and minimal invasiveness of TADs substantially expand clinical applications and improve the predictability of CAs [11]. Many researches have been conducted on MIA types, failures, optimal levels of placement, and optimal forces used for tooth movement in conventional fixed multibracket (FMB) therapy. Nevertheless, only a few CA strategies with an entirely distinct force application system have emerged [12, 13]. There are few published data regarding the selection of traction methods for elastics attached to microimplants and CAs[14]. Consequently, there is a lack of clinical recommendations available to orthodontists regarding the utilization of microimplants in conjunction with CAT for the purpose of molar distalization.

The finite element model (FEM) is a computerized numerical method that can be used to quantify initial tooth movement after force loading, allowing for a quantitative representation of a three-dimensional object. This method is commonly used in biomechanical research to analyse displacement and stress responses in various applications. Recently, FEM has been shown to be an effective tool for modelling tooth displacement patterns in orthodontics. Ayidaga et al. [15] analyzed the effect of different attachment configurations on the efficacy of upper maxillary molar bodily movement. Nevertheless, the simulation in that study was limited to a single tooth analyzed only in the sagittal plane. Rossini et al. [16] assessed the force system of the upper arch during second maxillary molar distalization with CAT and variable attachment settings in which no auxiliaries for anchorage reinforcement were used, and the sequential molar distalization process was not considered. Our previous study explored a series of biomechanic mechanisms and clinical problems concerning CAT [17–20], including the effects of upper-molar distalization using CA combined with Class II elastics [21].

This study was an innovative attempt to biomechanically evaluate the effective contribution ratio of molar distal movement in a 0.25-mm step distance during sequential distalization using CAT in combination with microimplants. This study also explored clinical guidelines for traction methods and elastic force magnitude selection.

## Methods

### Creating an original 3D model from cone-beam computed tomography (CBCT) data

Cone-beam computed tomography (CBCT) data (GE Healthcare, USA) were obtained from a Class II 24-year-old female patient with healthy craniofacial anatomy and complete dentition with third molars extracted. CBCT data were obtained previously for therapeutic purposes. The participant gave her informed consent for inclusion before participating in the study. The study was conducted following the tenets of the Declaration of Helsinki and was approved by the Ethics Committee of the Air Force Medical University in China (IRB-REV-2022079). The thickness of each CBCT slice was set as 0.15 mm, and 668 horizontal slices were reconstructed in total. The CBCT data were imported into Mimics 20.0 software (Materialise Software, Leuven, Belgium). A threshold procedure was used to create the mask layers of the maxilla and upper teeth. The original 3D model was rebuilt using the Calculate 3D command. The initial 3D models were optimized using Geomagic Studio 2014 (Raindrop GEOMAGIC, North Carolina, USA) software, and a surface model structure was created. The preliminary model for periodontal ligament (PDL), alveolar bone, microimplants, and attachments was built using the 3D mechanical drawing software NX 1911 (Siemens, Germany), as previously described [15, 20]. The PDL was reconstructed as a uniform layer by expanding the outside surface of the tooth roots by 0.25 mm. The maxilla bone moved inward after offset by 1.3 mm to construct a cancellous bone model; next, the cortical bone was built by subtracting cancellous from maxilla bone. Vertical rectangular attachments (2 × 3 × 1 mm) were constructed on the buccal surface of all the premolars for retention purposes, and a horizontal rectangular attachment (3 × 2 × 1 mm) was designed on the upper 2nd molars. The tooth crowns and attachments were extended outwards by 0.5 mm to simulate a CA appliance. A pair of microimplants (8 mm in length, 1.5 mm in diameter) was constructed and positioned between the second premolar and first molar at an angle of 60° with the occlusal plane and a height of 5 mm from the alveolar crest [22, 23].

### Creating submodels from the original 3D model

**Fig. 1:**
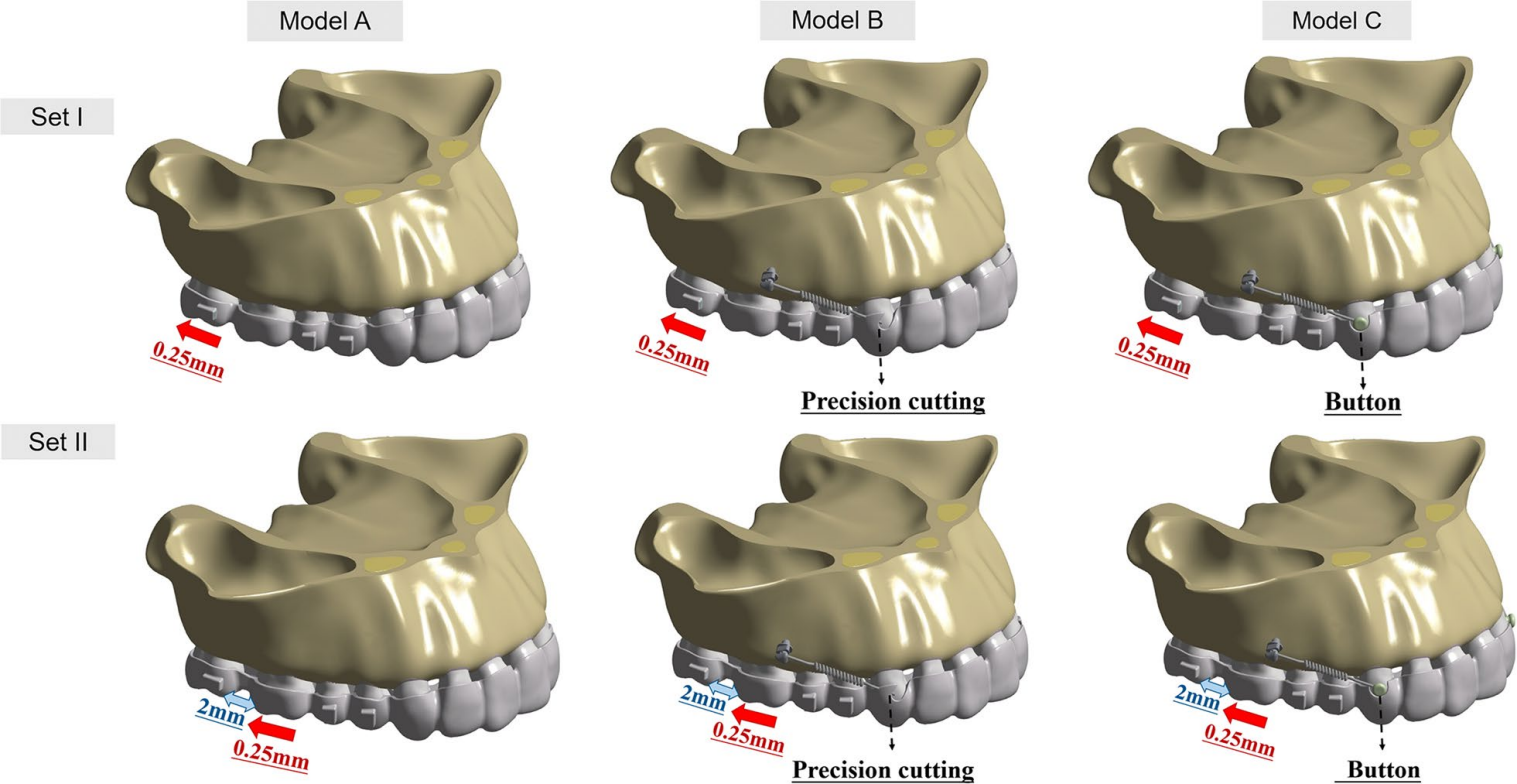
Model figures. Two group sets including six submodels were constructed. Group set I was created to simulate the initial distalization of the 2nd molar, whereas Group set II was built to model the initial distalization of the 1st molar after the 2nd molar had been distalized by 2 mm. Models A1 and A2 were control models simulating upper-molar distalization with clear aligners, and no anchorage reinforcement was used. Models B1 and B2 represent the upper-molar distalization combining transparent aligners with microimplants via buttons. Models C1 and C2 were designed to simulate upper-molar distalization using clear aligners in combination with microimplants attached by precision cuts

The original model and appliances were imported into ANSYS Workbench 2019 (Ansys, Pennsylvania, USA) to produce a 3D FE-based model. SOLID187, a 3D 10-node tetrahedral structural solid, was used. The material characteristics were consistent with those in prior studies [17, 20, 24, 25] (Table 1). All constructions were assumed to be made of linear, elastic, isotropic, and homogeneous materials. Two group sets with three submodels (Fig. 1) were created to simulate the simplified sequential molar distalization process using design inspiration drawn from prior work [26, 27]. Group set I was used to simulate 0.25 mm distal movement of the 2nd molars, whereas group set II was used to model 0.25 mm distal movement of the 1st molar after the 2nd molar had been moved distally by 2 mm. Control models A1 and A2 were used to simulate no anchorage reinforcement. Models B1 and B2 were featured with precision cuts. Precision cuts consisted of a hook on the clear aligner at the surface of canine. Models C1 and C2 had buttons (diameter of bottom surface: 3 mm, height: 1 mm) on the maxillary canines, with corresponding sections of the CA removed. The buttons and precision cuts were designed according to practical situations and clinical studies [14].

**Table 1:** Material properties

Material	Young’s modulus (MPa)	Poisson’s ratio
Tooth [17, 20, 24, 25]	1.96 × 10^4^	0.3
PDL[17, 20, 24, 25]	6.9 × 10^–1^	0.45
Cortical bone[17, 20, 24, 25]	1.37 × 10^4^	0.26
Cancellous bone[17, 20, 24, 25]	1.37 × 10^3^	0.3
Clear Aligner[17, 20, 24, 25]	5.28 × 10^2^	0.36
Attachments[17, 20, 24, 25]	1.25 × 10^4^	0.36
Buttons [25]	1.14 × 10^5^	0.35
micro-implants[25]	1.14 × 10^5^	0.35

### Boundary and contact conditions

**Fig. 2:**
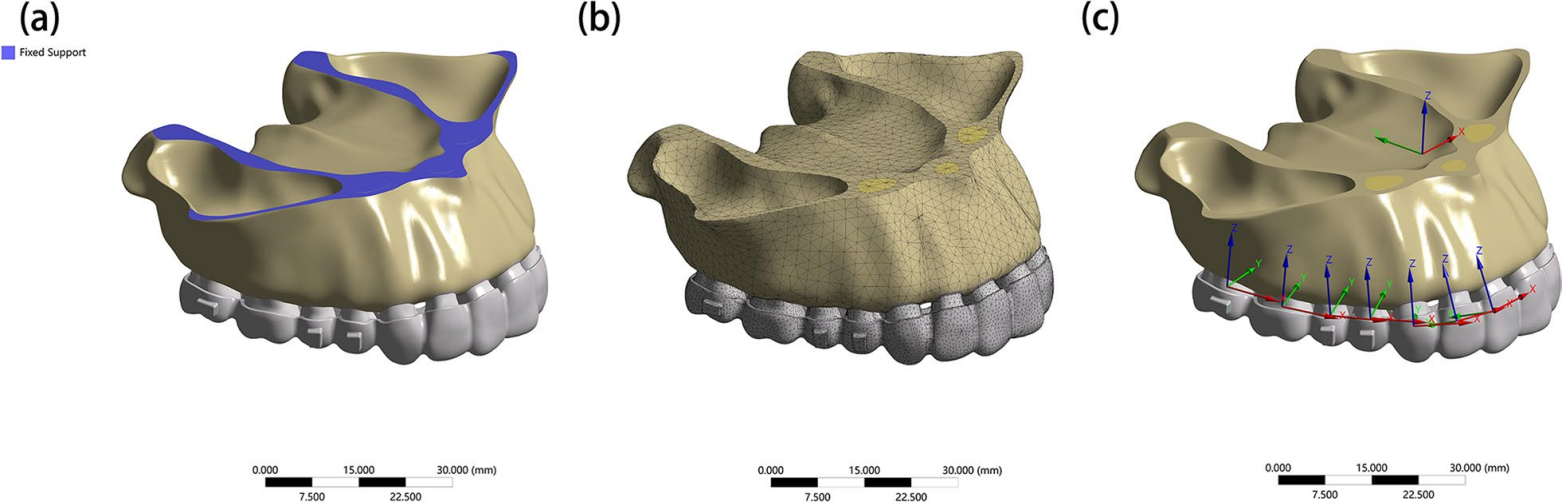
The boundary conditions, mesh figure, and coordinate systems. **a** The boundary conditions. The movement of temporal and maxilla bones was restricted for all degrees of freedom of the nodes at its superior region. **b** A figure showing the mesh. **c** Image showing the global and local coordinate systems

Regarding the boundary conditions (Fig. 2a), the movement of the temporal and maxilla bone was restricted to all degrees of freedom of the nodes in its superior area. Bonding contacts were established for interfaces of spongious-cortical bone, cortical bone-PDL, bone microimplants, PDL tooth, tooth buttons, and tooth attachment. Such bonding prevents any movement between contact surfaces. Furthermore, the connections between the adjacent teeth were assumed to have no separation from their interfaces. A friction-based condition with a friction coefficient of 0.2 was established in the contact surfaces between the CA and the tooth crown surface.

### Loading method

Static loading was used in the FEM simulation. The step distance of molar distal movement was set to be 0.25 mm [3]. In group set I, the 2nd molar was subjected to a distal movement of 0.25 mm to create a loaded condition CA. The loading force was then applied by the mismatch between the CA and the initial dentition. Subsequently, in group set II, the 2nd molar was displaced distally by 2 mm to reach a target position, followed by the 1st molar underwent a distal movement of 0.25 mm distally to establish a loading condition CA [17]. Finally, in models B and C, a series force of 100, 150, and 200 g was applied by a spring attached to the microimplants and the buttons or precision cuts on each side [26]. NiTi springs are employed for the purpose of simulating the implementation of elastic traction.

### Coordinate systems and outcomes

The FE mesh was divided by the discretization process (Fig. 2b). The nodes and linear elements of each submodel are shown in Table 2. For reference, two coordinate systems were established [24] (Fig. 2c). The global coordinate system was defined for the whole dentition, with the coronal plane represented by the x-axis (+ left, − right); the sagittal plane denoted by the y-axis (+ posterior, − anterior), and the vertical plane denoted by the z-axis (+ superior, − inferior). The local coordination system was defined for each tooth as follows: the x-axis (+ mesial, − distal), the y-axis (+ lingual, − buccal), and the z-axis (+ apical, − crown). The displacement tendencies of both the entire dentition and individual teeth, as well as the hydrostatic stress on the periodontal ligament and the von Mises stress on the alveolar bone were calculated by ANSYS Workbench 2019 (Ansys, Pennsylvania, USA)[28, 29]. Reference points of anterior teeth were tooth cusp and root apex, and that of the posterior teeth were mesio-buccal cusp and palatal root apex. According to the local coordination system, the contribution ratios of molar distal movement and anchorage teeth mesial movement were calculated using the formula |x-axis displacement|/0.25 × 100%. Other undesired movements, such as tipping and rotation, occupied the remaining percentage.

**Table 2:** Nodes and elements

	A1	A2	B1	B2	C1	C2
Nodes	694,964	704,124	709,482	719,254	711,376	720,443
Elements	391,609	397,830	399,748	406,462	400,825	407,009

## Results

### The effective contribution ratio of molar distal movement at a 0.25-mm step distance

**Fig. 3:**
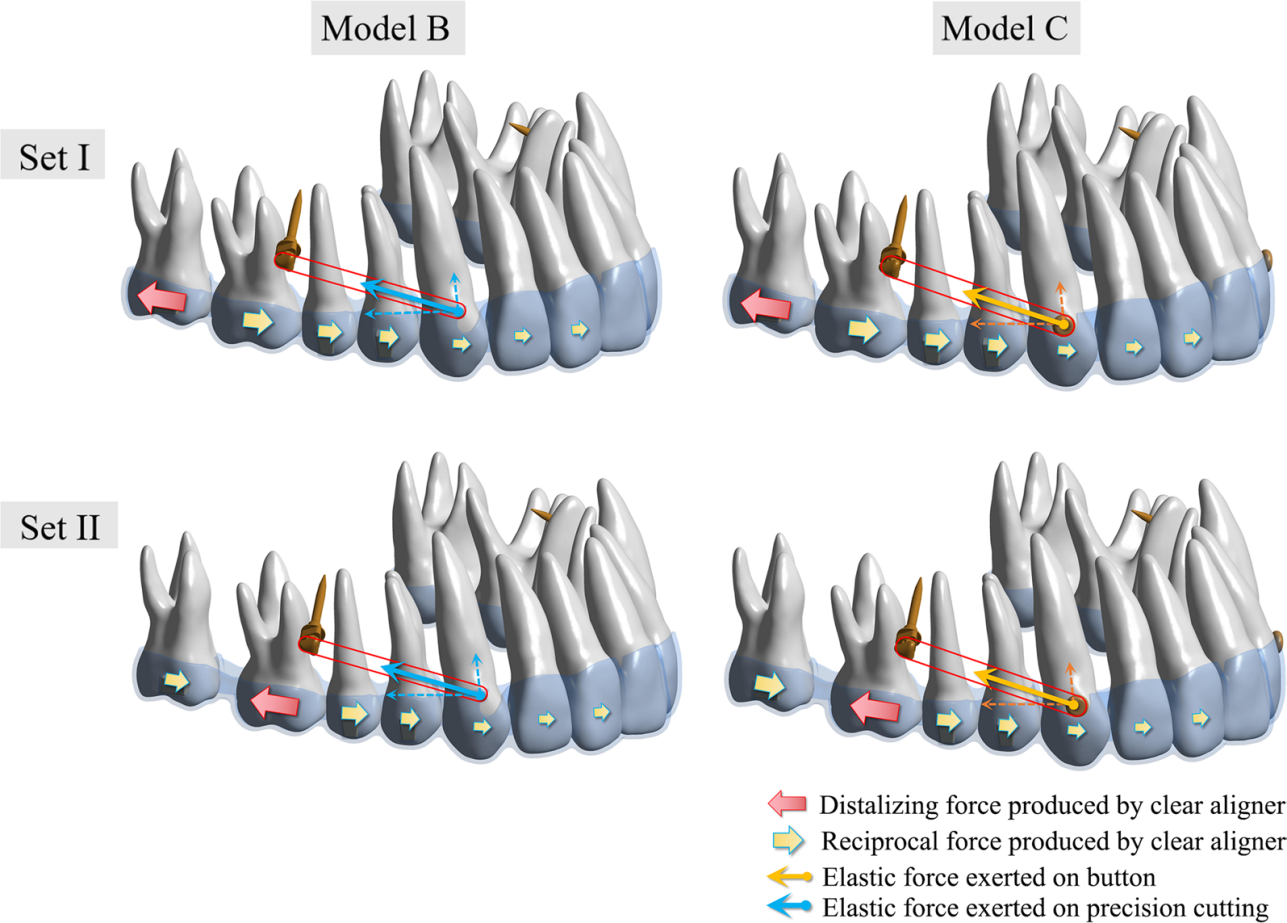
The force loading system. The pushing force produced by the CA varied from group set I to set II. When the upper 2nd molar was distalized, the CA produced a mesial reciprocal force on the anchorage teeth. When moving the 1st molar distally, the pushing force exerted a reciprocal force on the 2nd molars and other anchorage teeth. In model B, the elastic force was exerted on the CA, whereas in model C, it was applied directly on the upper canines by button

**Fig. 4:**
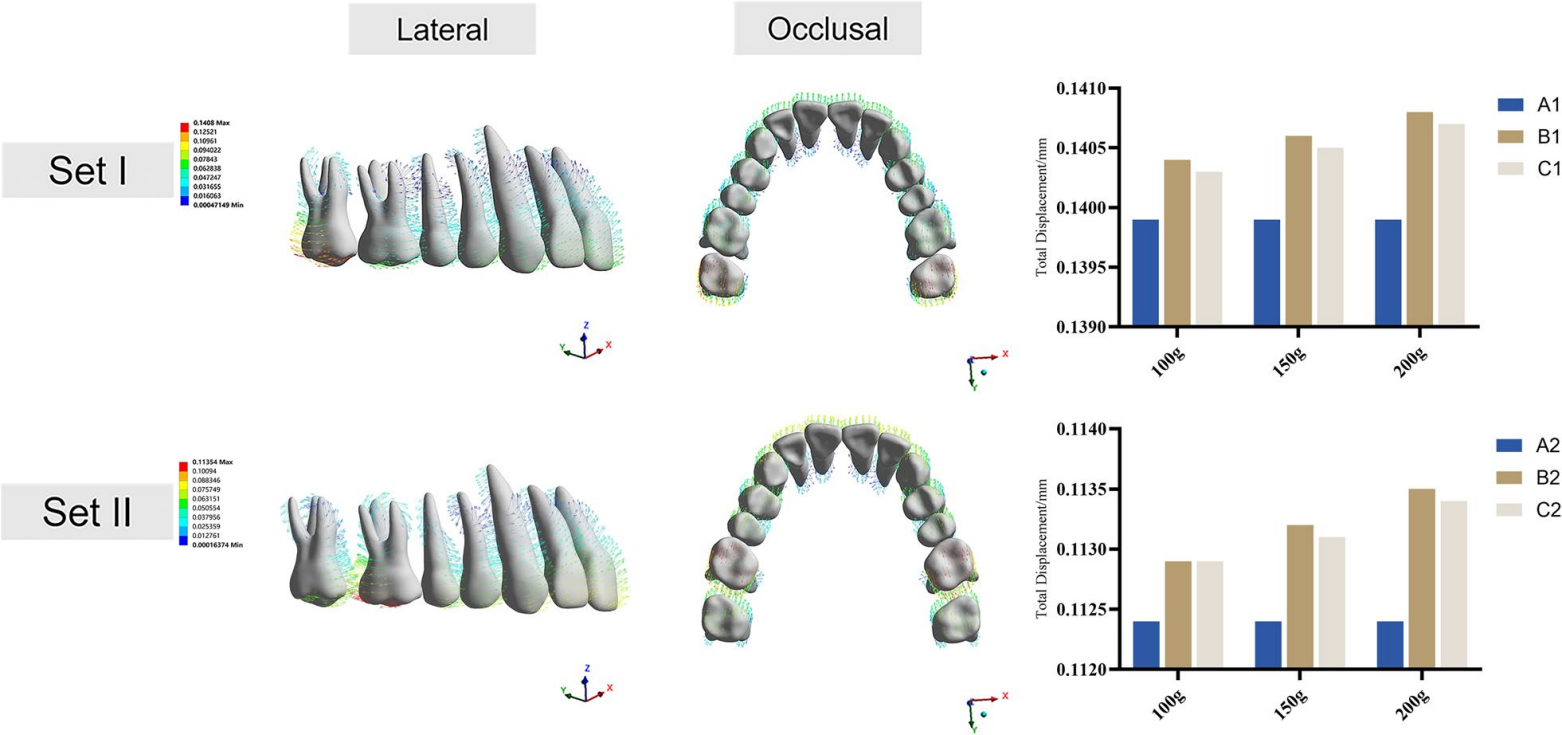
Total displacement of the maxillary dentition. Set I, initial distalization of the 2nd molar; set II, initial distalization of the 1st molar. Model A, control model without anchorage reinforcement; model B, microimplants attached to the tooth by precision cuts; model C, microimplants attached to the aligner by a button. Vector diagrams from the lateral and occlusal views show the displacement direction of the whole maxillary dentition. Histograms display the total displacement values of different models in two group sets with various traction forces (mm). The coordinate system was based on the entire dentition (global coordinate system). The x-axis represents the coronal plane (+ left, − right), the y-axis represents the sagittal plane (+ posterior, − anterior), and the z-axis represents the vertical plane (+ superior, − inferior)

**Fig. 5:**
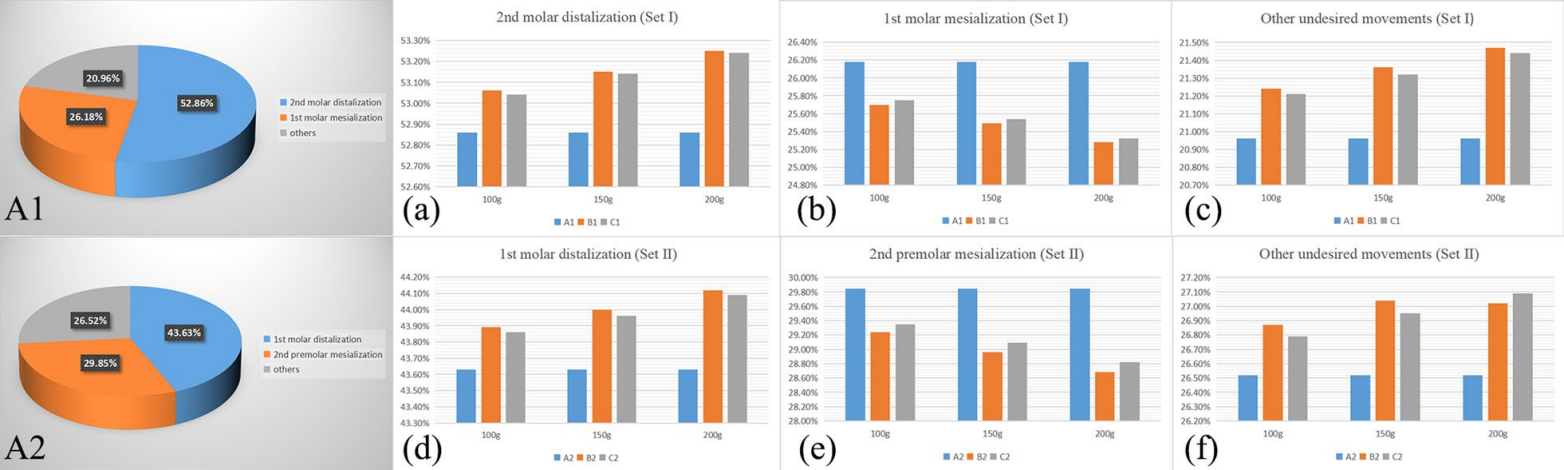
The effective contribution ratio of molar distal movement at a 0.25 mm step distance. The pie charts show the contribution ratio of molar distalization, anchorage teeth mesialization to the 0.25 mm step distance, and the other percentage were occupied by buccal tipping and rotation. A1, model A1; A2, model A2. Subimage **a** represents the contribution ratio of 2nd molar distalization in group set I with various traction forces. Subimage **b** represents the contribution ratio of 1st molar mesialization during the 2nd molar distalization. Subimage **c** represents the contribution ratio of the other undesired movements. Subimage **d** represents the contribution ratio of 1st molar distalization in group set II. Subimage **e** represents the contribution ratio of 2nd premolar mesialization during the 1st molar distalization. Subimage **f** represents the percentage of other undesired movements

Figure 3 illustrates the force‒loading systems of CAs and MIAs. In model B, the elastic force is exerted on the CA through precision cuts, whereas in model C, it is applied directly to the upper canines by a button. The pushing force produced by the CA varied from group set I to set II. When the upper 2nd molar was distal moved, the CA produced a mesial reciprocal force on the anchorage teeth. When the 1st molar was underwent distal movement, the CA exerted reciprocal force on the 2nd molars and other anchorage teeth. The total displacement of the maxillary dentition was recorded according to the global coordinate system. The reciprocal force caused the distal moving molars and the anchorage teeth to move in opposite directions in all models (Fig. 4). The highest total displacement tendency was in model B1, with precision cuts and 200 g traction force (0.1408 mm). In contrast, the lowest total displacement tendency was in model A2 (0.1124 mm) without microimplants for anchorage reinforcement. Figure 5 elucidated the effective contribution ratio of the 0.25-mm step distance. In model A1, the distal displacement of the 2nd molars accounted for 52.86%, while the mesial displacement of the 1st molars accounted for 26.18%. The remaining 20.96% were occupied by tipping and rotation. In model A2, the distal displacement of the 1st molars accounted for 43.63%. The mesial displacement of the 2nd premolars accounted for 29.85%, and the remaining 26.52% was occupied by buccal tipping and rotation. Compared with model A, models B and C had higher effective contribution ratio of molar distalization and lower ratio of anchorage teeth mesialization with anchorage reinforcement by microimplants. Model B had a higher effective contribution ratio (set I: 100 g, 53.06%; 150 g, 53.15%; and 200 g, 53.25%; set II: 100 g, 43.89%; 150 g, 44%; and 200 g, 44.12%) than model C (set I: 100 g, 53.04%; 150 g, 53.14%; and 200 g, 53.24%; set II: 100 g, 43.86%; 150 g, 43.96%; and 200 g, 44.09%). The effective contribution ratio occupied by molar distalization increased with increasing traction force, whereas the ratio of anchorage tooth mesialization decreased. However, the percentage of tipping and rotation increased slightly. Furthermore, the effective contribution ratio of the 1st molars in set I was lower than that of the 2nd molars in set II.

### 3D displacement of the posterior teeth

**Fig. 6:**
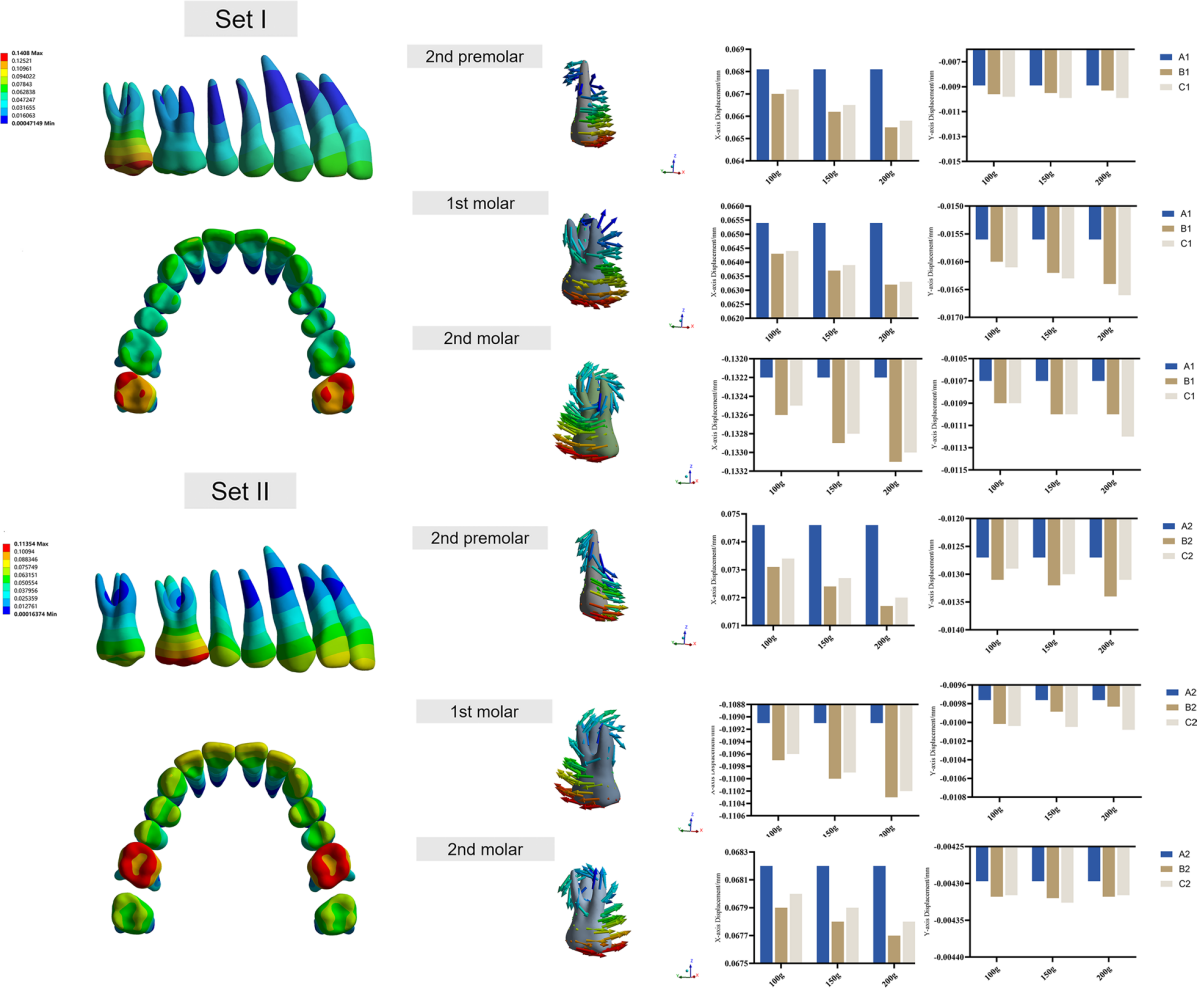
Three-dimensional displacement of the posterior teeth. In color maps, the red color shows the maximum displacement areas, and the blue color shows the minimum displacement areas. Vector diagrams show initial displacement patterns of 2nd premolars, 1st molars, and 2nd molars. Histograms present the x-axis and y-axis displacement values (mm). The coordinate system was centered on each tooth (local coordinate system); the positive value for the x-axis represents the mesial surface of the teeth, and the positive value for the y-axis represents the palatal surface of the teeth

The 3D displacement values of the posterior teeth are summarized in Additional file 1 and illustrated in Fig. 6. The displacement direction of the posterior teeth was mainly along the mesiodistal direction. In group set I, the 2nd molars were distally moved with a distobuccal tipping tendency. Due to the reciprocal force produced by the CA, the 1st molars and premolars were moved mesially with a mesiobuccal tipping tendency. In group set II, the 1st molars moved distally, with a distobuccal tipping tendency. Simultaneously, the premolars moved mesially with a mesiobuccal tipping tendency. Based on the vector diagrams, the center of rotation can be observed at the apical direction of the root trifurcation of the molars. Notably, the 2nd molars, displaced distally by 2 mm at the target position, also had a mesiopalatal movement tendency with the reciprocal force produced by the 1st molar distalization, which can be defined as a relapse phenomenon. Compared with model A, models B and C had a higher distalization tendency of molars, lower mesialization tendency of anchorage teeth, and lower relapse tendency. Model B had superior anchorage reinforcement than model C. As the magnitude of the traction force increased, the reinforcement of the anchorage became increasingly conspicuous.

### 3D displacement of anterior teeth

**Fig. 7:**
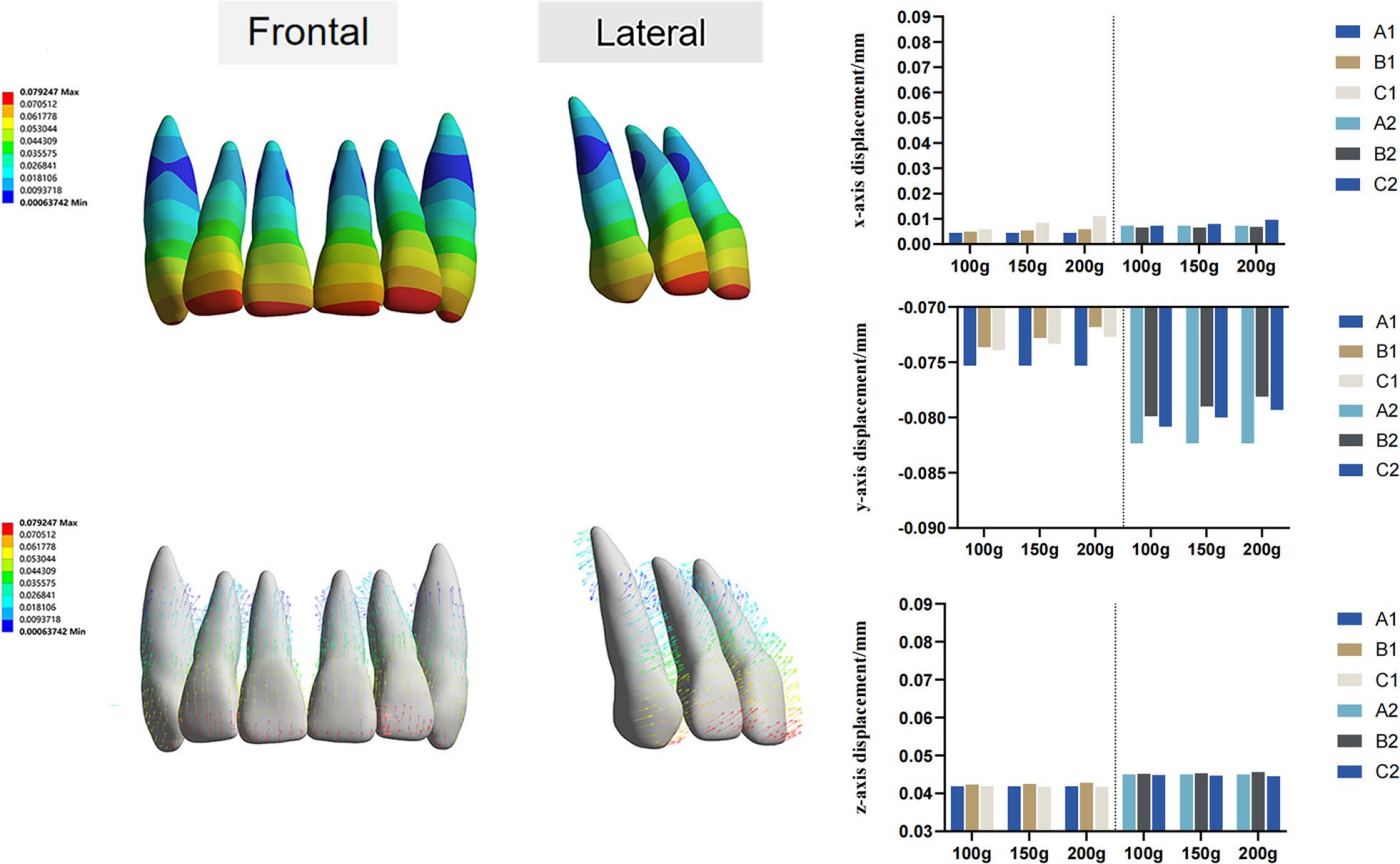
Three-dimensional displacement of the anterior teeth. Color maps and vector diagrams show the initial displacement patterns of central incisors, lateral incisors, and canines. Histograms present three-dimensional displacement values of different models in two group sets with various traction forces (mm). The coordinate system was centered on each tooth (local coordinate system)

The movement direction of the anterior teeth was mainly on the y-axis (Additional file 2). The anterior teeth demonstrated a mesiolabial tipping and intrusion tendency in all models (Fig. 7). The center of rotation was at the middle third of the root. The undesired movement of the incisors and canines was reduced in models B and C compared to that in model A, as microimplants were utilized for anchorage enhancement. Model B had a more significant anchorage control than model C for incisors, whereas model C had somewhat better anchorage control for canines. Furthermore, when the traction force increased, so did the anchorage enhancement. Among the anterior teeth, the lateral incisors were labial tipped to a greater extent on the y-axis, which was adequately managed in model B. The labial tipping tendency of the anterior teeth increased with the distalization of the 1st molar in group set II, indicating increased anchorage loss.

### PDL hydrostatic stress and alveolar bone von Mises stress

**Fig. 8:**
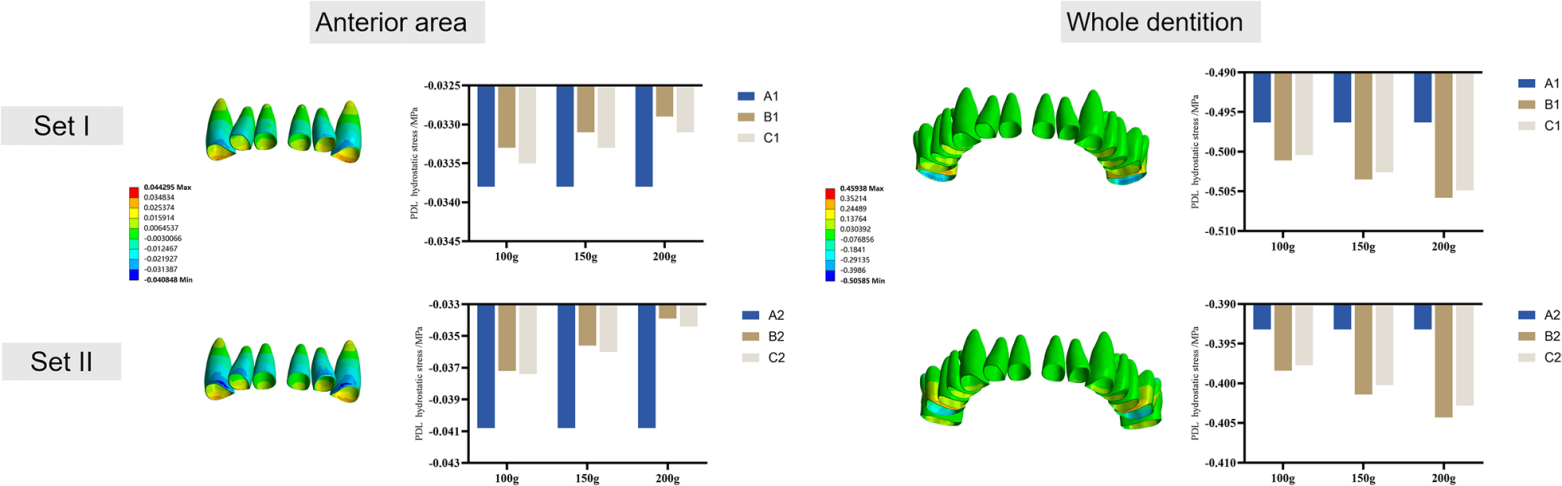
PDL hydrostatic stress. The color maps show the buccal view of the PDL at the anterior area and the whole dentition. Histograms present compressive pressures of PDL hydrostatic stress (MPa)

**Fig. 9:**
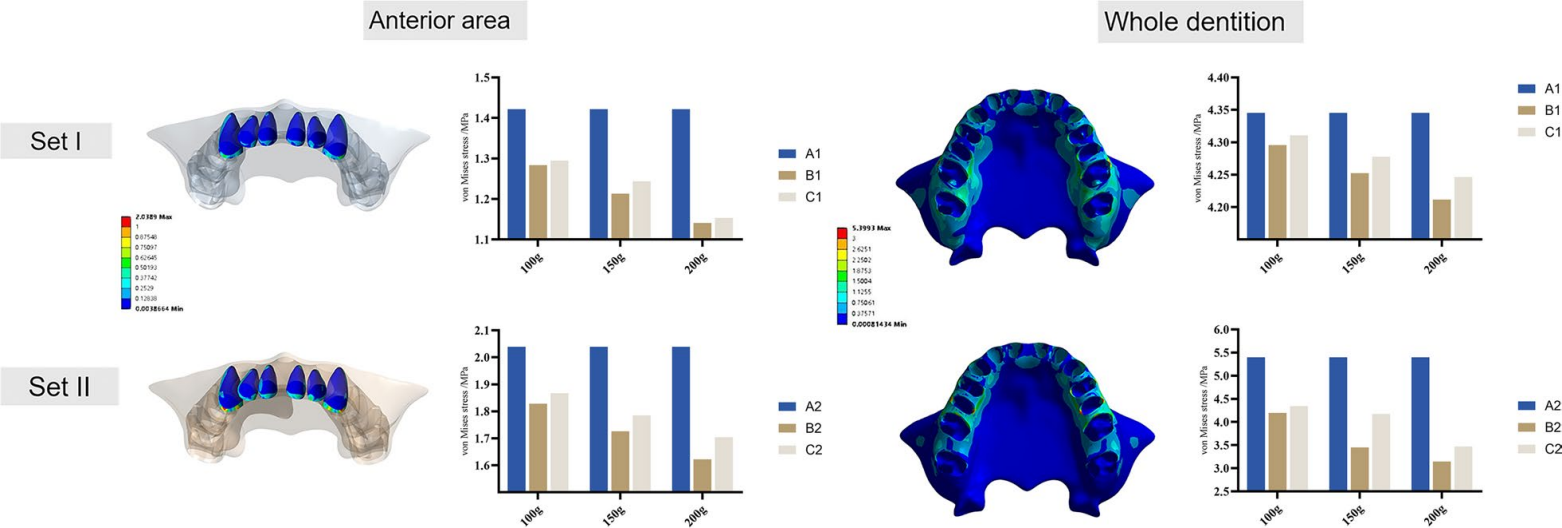
von Mises stress of the alveolar bone. The color maps show the buccal view of the alveolar bone at the anterior area and the whole dentition. Histograms present the von Mises stress of the alveolar bone (MPa)

PDL hydrostatic pressure (Fig. 8) and von Mises stress of the alveolar bone (Fig. 9) were recorded using the anterior anchorage unit and the entire dentition as observation units, respectively. The highest compressive stress of the PDL in the anterior area was concentrated on the labial cervical region and the apex of the upper incisors. The highest compressive stress in canines was concentrated on the mesiobuccal cervical and apex areas. The highest compressive stress was on the distal cervical surface of the distalizing molars considering the entire dentition. Model A underwent the highest compressive PDL stress at the anterior area and the lowest at the molars. The lowest stress at the anterior area and highest stress at the molars were both observed in model B. The von Mises stress of the alveolar bone was correspondingly concentrated at the labial alveolar crest in the anterior area and the alveolar bone around the distalizing molars.

## Discussion

CAT has been found to provide unique advantages for molar distalization, with aesthetic and comfort benefits and superior vertical control. Nonetheless, labial tipping of the anterior teeth was detected to variable degrees in patients who did not use auxiliaries other than composite attachments throughout the molar distalization treatment because of the reciprocal force produced by molar distalization [30]. Therefore, anchorage loss was inevitable during molar distalization using CAT. Pertinently, microimplants are widely used in FMB for anchorage reinforcement, providing more personalized options for tooth movement across a broader range of malocclusions. However, the therapy using CA in conjunction with microimplants remains unknown. There is no predictable method to follow when utilizing CA if the distalization distance is larger than 3 mm [31]. Because of the limits of the removable thermoplastic materials of CAs and special force application systems, the setup in the CA software could not accurately reflect the actual movement of the teeth. Molar distalization efficiency using untreated teeth as reference points for superimposition was evaluated in recent studies, whereas the mesial movement of anchorage teeth could not be ignored in reality[3]. Thus, more predictable guidance for using MIA in combination with CAT is needed.

This was the first study to biomechanically calculate the effective contribution ratio of molar distal movement during sequential distalization using CA with microimplants. From our observation, the distalization force caused the distalizing molars to be distobuccally tipped, while the reciprocal force caused the anchorage teeth to be mesiobuccally tipped [32–34]. Without anchorage reinforcement, the distal displacement accounted for 52.86% of the 0.25-mm step distance during the 2nd molar distalization. In comparison, the mesial displacement of the 1st molars accounted for 26.18%, and the remaining percentage was occupied by tipping and rotation. There was an increased effective contribution ratio of molar distalization and decreased mesialization of the anchorage teeth with the anchorage reinforcement provided by microimplants. Furthermore, microimplants attached to the aligner by precision cuts demonstrated superior enhancement for the effective contribution ratio and anchorage reinforcement by loading and transmitting the anchorage force to the whole dentition directly by the aligner. In contrast, microimplants attached to buttons loaded anchorage force onto the canines. This was transmitted by the squeezing force between the neighbouring teeth and weakened by the gap between the teeth during transmission.

The displacement pattern of teeth is a function of the relationship between the CRes and the action of the force. As reported by Gandhi et al. [35], the CRes of molars were measured at the geometric center of the buccal surface of the molar and the trifurcation of the molar roots. The centers of rotation varied with the moment-to-force (M/F) ratio. The center of rotation in this study can be observed at the apical direction of the root trifurcation of the distalized molars. The force system includes the external force applied to the teeth by the CA and the internal force transferred between the adjacent teeth. The distalizing force produced by the CA was applied to the crowns of the teeth coronally to the CRes of the molars, therefore facilitating the distal crown tipping of the molars [36]. The contact area of the adjacent surface of premolars and molars is on the buccal side of tooth surfaces that is tipping them buccally. As a result, the distobuccal tipping of the molars causes extrusion of the palatal tooth cusps and rotation of the teeth. The buccal tipping and rotation were responsible for approximately 20.96% of the step distance during the 2nd molar distalization without skeletal anchorage. Conversely, the undesired displacement of molar tipping and rotation increased slightly with anchorage reinforcement by microimplants. Clinical practitioners could place some attachments on the palatal surfaces to counteract the buccal tipping force and design some degree of crown-lingual overcorrection.

The anterior teeth demonstrated a mesiolabial tipping tendency. This was due to the fact that the point of force application passed above the CRes of the anterior teeth and produced a tipping moment. Consequently, the PDL hydrostatic pressure was mainly concentrated on the labial cervical region and root apex for the upper incisors and the mesiobuccal cervical and root apices of canines. As reported in previous investigations [37, 38], if the PDL hydrostatic pressure exceeds the capillary pressure in the area, the vessels collapse, and blood flow impairs the area, increasing the risks of root resorption. Although the incidence is lower with CAT than with fixed appliances, root resorption cannot be avoided, particularly for incisors [39].

Additionally, the corresponding higher stress on the labial alveolar crest of the anterior area can lead to an increased risk of bone defects, such as bone fenestration and dehiscence. These features are frequently encountered in clinical practice, particularly concerning incisors [40]. The labial tipping of anterior teeth was effectively controlled with a low PDL hydrostatic stress value and low von Mises stress of the alveolar bone with microimplants for anchorage reinforcement, minimizing the risk of root resorption and bone defects in the anterior area. Precision cuts might be a superior method when better distalization efficiency and anchorage reinforcement are needed. For example, labial tipping of upper incisors is undesirable when molars are meant to be distalized for a long distance in Class II malocclusion division 1 with a deep overjet, which is commonly linked with labial tipping of the incisors and weak cortical bone in the anterior area. In such cases, a heavy magnitude of traction force might also play an essential role in anchorage reinforcement. However, in some circumstances, such as Class II division two malocclusion with a deep bite and retroclined incisors, anterior tooth labial tipping is regarded as a desirable movement. In this case, MIA connected by buttons with a light force would be a more appropriate option. Moreover, buttons were more suitable when the canines required more anchorage control. However, regardless of the traction strategy chosen, some anchorage loss still existed. Therefore, an optimized torque design should be proposed in future studies to facilitate anchorage control management.

Several staging patterns of molar distalization with CAs have been reported, including sequential and simultaneous distalizations. Sequential molar distalization is the most commonly used method in clinical practice [41]. Ojima et al. [27] deduced from clinical observation that sequential distalization could protect anterior anchorage to the maximum extent. Sujaritwanid et al. [42] found that sequential distalization was the most efficient treatment approach for obtaining controlled distalization of a molar, with the advantage of applying relatively low forces with reduced dental undesirable effects. This study analyzed sequential molar distalization using CAT. We found that the 2nd molar had a mesial movement tendency after being distal placed to the target position during the distalization of the 1st molar. This relapse phenomenon might be explained by the mesial reciprocal force produced by the distal movement of the 1st molar. Therefore, orthodontists should maintain a pretreatment molar position during sequential distalization. The effective contribution ratio of the 1st molars was lower than that of the 2nd molars, with 43.63% distal displacement, 29.85% mesial displacement of the anchorage teeth, and 26.52% of the others. This result is in accordance with the conclusions of Saif et al. [30]. The effective contribution ratio of the 1st molar increased, and the relapse tendency of the 2nd molars also decreased with anchorage reinforcement by microimplants. The anchorage loss of anterior teeth also increased during the distalization of the 1st molar. We inferred that with the reduction in the arm of force between the anterior anchorage and the distalized molar, the required force magnifies, thus resulting in increased anchorage loss. Therefore, enhanced protection of anchorage is required during the sequential distalization process.

Force magnitude from 100 to 200 g was reported as a safe force application range [22]. We found that the effective contribution ratio of molar distalization and anchorage reinforcement increased with increased force magnitude. In addition, the 2nd molar relapse tendency was also reduced with an increase in force. However, an undesired movement of molar tipping, rotation, and stress on molars increased as the force increased. Park et al. [43] also considered that a large magnitude of force, up to 200 g, was not excessive when used for the distal movement of molars.

Nevertheless, this study had some limitations. First, the thickness of the PDL was assumed to be uniform, whereas in reality, it has an hourglass shape with the narrowest zone at the mid-root level [44]. The material properties of PDL have historically been controversial, with an elastic modulus ranging from 0.01 to 100 MPa. Some scholars insist on the nonlinearity of its elastic modulus. In contrast, it has been shown that nonlinearity mainly affects the magnitude of the stress, not the actual movement pattern of the teeth [45]. Second, this static analysis provides only a theoretical initial movement tendency. At the same time, the exact clinical outcomes are influenced by the accumulative effects of alternate force and bone remodeling. Last, multiple biological factors are involved in tooth movement, including long-term contact of the root with cortical bone, sinus proximity or invagination, root morphology, and metabolism of the periodontium [28]. Therefore, the clinical translation of the conclusions should be taken with caution. We suggest that future clinical studies use more stable structures, such as palatal rugae registration, instead of anchorage teeth, as reference points.

## Conclusion

This study aimed to biomechanically analyze the effective contribution ratio of molar distal movement at a 0.25-mm step distance during sequential distalization using clear aligner with microimplant. The findings revealed that the distalized molars exhibited distobuccal tipping, while the anchorage teeth experienced mesiobuccal tipping due to the reciprocal force. Consequently, the effective contribution ratio of molar distal movement decreased, and anchorage consumption increased in the process of sequential distalization. These results suggest the necessity for enhanced anchorage protection in such procedures. The effective contribution ratio of molar distalization increased, accompanied by a reduction in anchorage consumption due to the reinforcement provided by microimplants. Nevertheless, there was a slight increase in undesired molar tipping and rotation. Additionally, precision cuts demonstrated a superior effective contribution ratio and anchorage reinforcement for incisors when compared to buttons.

### Supplementary Information

**Additional file 1**. Three-dimensional displacement values for the posterior teeth (mm).

**Additional file 2**. Three-dimensional displacement values for the anterior teeth (mm).

## Data Availability

The authors confirm that the data supporting the findings of this study are available within the article and its supplementary materials.
