# Supplementary material for: Effective contribution ratio of the molar during sequential distalization using clear aligners and micro-implant anchorage: a finite element study
Source: Prog Orthod. 2023 Oct 9;24:35. doi: 10.1186/s40510-023-00485-0 (PMC10560653; doi:10.1186/s40510-023-00485-0)
Supplement: Supplementary file 1 — Additional file 1. Three-dimensional displacement values for the posterior teeth (mm). [file 40510_2023_485_MOESM1_ESM.docx]

**Supplementary file 1.** Three-dimensional displacement values for the posterior teeth (mm).

| Maxillary | Force magnitudes | Directions | x-axis | | | | | | y-axis | | | | | | z-axis | | | | | |
| --- | --- | --- | --- | --- | --- | --- | --- | --- | --- | --- | --- | --- | --- | --- | --- | --- | --- | --- | --- | --- |
|  |  | Groups | Set I | | | Set II | | | Set I | | | Set II | | | Set I | | | Set II | | |
|  |  | Models | A | B | C | A | B | C | A | B | C | A | B | C | A | B | C | A | B | C |
| First premolar | 100g | Crown | 0.0669 | 0.0653 | 0.0656 | 0.0656 | 0.0642 | 0.0646 | -0.0222 | -0.0215 | -0.0214 | -0.0297 | -0.0286 | -0.0284 | 0.0304 | 0.0294 | 0.0298 | 0.0264 | 0.0254 | 0.0257 |
|  |  | Root | -0.0173 | -0.0164 | -0.0167 | -0.0197 | -0.0192 | -0.0194 | 0.0092 | 0.0089 | 0.0089 | 0.0097 | 0.0094 | 0.0093 | -0.0058 | -0.0056 | -0.0054 | -0.0116 | -0.0117 | -0.0115 |
|  | 150g | Crown | 0.0669 | 0.0646 | 0.0649 | 0.0656 | 0.0636 | 0.0640 | -0.0222 | -0.0210 | -0.0210 | -0.0297 | -0.0279 | -0.0278 | 0.0304 | 0.0290 | 0.0295 | 0.0264 | 0.0249 | 0.0254 |
|  |  | Root | -0.0173 | -0.0161 | -0.0165 | -0.0197 | -0.0190 | -0.0192 | 0.0092 | 0.0088 | 0.0088 | 0.0097 | 0.0091 | 0.0092 | -0.0058 | -0.0054 | 0.0295 | -0.0116 | -0.0117 | -0.0114 |
|  | 200g | Crown | 0.0669 | 0.0638 | 0.0642 | 0.0656 | 0.0630 | 0.0634 | -0.0222 | -0.0204 | -0.0206 | -0.0297 | -0.0271 | -0.0271 | 0.0304 | 0.0286 | 0.0293 | 0.0264 | 0.0244 | 0.0250 |
|  |  | Root | -0.0173 | -0.0157 | -0.0162 | -0.0197 | -0.0188 | -0.0191 | 0.0092 | 0.0086 | 0.0087 | 0.0097 | 0.0088 | 0.0090 | -0.0058 | -0.0053 | -0.0050 | -0.0116 | -0.0117 | -0.0113 |
| Second premolar | 100g | Crown | 0.0681 | 0.0670 | 0.0672 | 0.0746 | 0.0731 | 0.0734 | -0.0089 | -0.0096 | -0.0098 | -0.0127 | -0.0131 | -0.0129 | 0.0174 | 0.0294 | 0.0298 | 0.0063 | 0.0254 | 0.0257 |
|  |  | Root | -0.0211 | -0.0204 | -0.0205 | -0.0222 | -0.0215 | -0.0216 | 0.0019 | 0.0015 | 0.0015 | 0.0242 | 0.0247 | 0.0244 | -0.0145 | -0.0144 | -0.0144 | -0.0273 | -0.0131 | -0.0269 |
|  | 150g | Crown | 0.0681 | 0.0662 | 0.0665 | 0.0746 | 0.0724 | 0.0727 | -0.0089 | -0.0095 | -0.0099 | -0.0127 | -0.0132 | -0.0130 | 0.0174 | 0.0167 | 0.0169 | 0.0063 | 0.0055 | 0.0057 |
|  |  | Root | -0.0211 | -0.0200 | -0.0201 | -0.0222 | -0.0211 | -0.0213 | 0.0019 | 0.0015 | 0.0015 | 0.0242 | 0.0249 | 0.0245 | -0.0145 | -0.0142 | -0.0142 | -0.0273 | -0.0266 | -0.0267 |
|  | 200g | Crown | 0.0681 | 0.0655 | 0.0658 | 0.0746 | 0.0717 | 0.0720 | -0.0089 | -0.0093 | -0.0099 | -0.0127 | -0.0134 | -0.0131 | 0.0174 | 0.0165 | 0.0168 | 0.0063 | 0.0052 | 0.0054 |
|  |  | Root | -0.0211 | -0.0195 | -0.0197 | -0.0222 | -0.0208 | -0.0210 | 0.0019 | 0.0015 | 0.0015 | 0.0242 | 0.0252 | 0.0245 | -0.0145 | -0.0139 | -0.0140 | -0.0273 | -0.0264 | -0.0265 |
| First molar | 100g | Crown | 0.0654 | 0.0643 | 0.0644 | -0.1091 | -0.1097 | -0.1096 | -0.0156 | -0.0160 | -0.0161 | -0.00976 | -0.01001 | -0.01003 | 0.0154 | 0.0286 | 0.0293 | -0.0496 | 0.0244 | 0.0250 |
|  |  | Root | -0.0137 | -0.0127 | -0.0128 | 0.0414 | 0.0417 | 0.0417 | 0.0040 | 0.0040 | 0.0040 | 0.01044 | 0.01038 | 0.01045 | -0.0367 | -0.0366 | -0.0367 | 0.0435 | 0.0438 | 0.0437 |
|  | 150g | Crown | 0.0654 | 0.0637 | 0.0639 | -0.1091 | -0.1100 | -0.1099 | -0.0156 | -0.0162 | -0.0163 | -0.00976 | -0.00988 | -0.01004 | 0.0154 | 0.0143 | 0.0145 | -0.0496 | -0.0500 | -0.0500 |
|  |  | Root | -0.0137 | -0.0123 | -0.0124 | 0.0414 | 0.0418 | 0.0418 | 0.0040 | 0.0041 | 0.0041 | 0.01044 | 0.01041 | 0.01049 | -0.0367 | -0.0366 | -0.0367 | 0.0435 | 0.0439 | 0.0438 |
|  | 200g | Crown | 0.0654 | 0.0632 | 0.0633 | -0.1091 | -0.1103 | -0.1102 | -0.0156 | -0.0164 | -0.0166 | -0.00976 | -0.00983 | -0.01007 | 0.0154 | 0.0140 | 0.0142 | -0.0496 | -0.0502 | -0.0501 |
|  |  | Root | -0.0137 | -0.0119 | -0.0120 | 0.0414 | 0.0419 | 0.0419 | 0.0040 | 0.0041 | 0.0041 | 0.010441 | 0.010430 | 0.010541 | -0.0367 | -0.0366 | -0.0367 | 0.0435 | 0.0440 | 0.0440 |
| Second molar | 100g | Crown | -0.1322 | -0.1326 | -0.1325 | 0.0682 | 0.0679 | 0.0680 | -0.0107 | -0.0109 | -0.0109 | -0.00430 | -0.00432 | -0.00432 | -0.0561 | -0.0561 | -0.0561 | 0.0282 | 0.0281 | 0.0281 |
|  |  | Root | 0.0590 | 0.0592 | 0.0592 | -0.0276 | -0.0274 | -0.0274 | 0.0117 | 0.0117 | 0.0117 | 0.00278 | 0.00271 | 0.00272 | 0.0441 | 0.0444 | 0.0443 | -0.0232 | -0.0230 | -0.0231 |
|  | 150g | Crown | -0.1322 | -0.1329 | -0.1328 | 0.0682 | 0.0678 | 0.0679 | -0.0107 | -0.0110 | -0.0110 | -0.00430 | -0.00432 | -0.00433 | -0.0561 | -0.0562 | -0.0562 | 0.0282 | 0.0280 | 0.0281 |
|  |  | Root | 0.0590 | 0.0593 | 0.0593 | -0.0276 | -0.0273 | -0.0274 | 0.0117 | 0.0117 | 0.0116 | 0.00278 | 0.00268 | 0.00268 | 0.0441 | 0.0445 | 0.0444 | -0.0232 | -0.0230 | -0.0230 |
|  | 200g | Crown | -0.1322 | -0.1331 | -0.133 | 0.0682 | 0.0677 | 0.0677 | -0.0107 | -0.0110 | -0.0112 | -0.00430 | -0.00432 | -0.00432 | -0.0561 | -0.0562 | -0.0562 | 0.0282 | 0.0280 | 0.0280 |
|  |  | Root | 0.0590 | 0.0594 | 0.0594 | -0.0276 | -0.0273 | -0.0273 | 0.0117 | 0.0117 | 0.0117 | 0.00278 | 0.00267 | 0.00265 | 0.0441 | 0.0446 | 0.0446 | -0.0232 | -0.0229 | -0.0230 |

The coordinate system was centered on each tooth( local coordinate system). A positive value on the x-axis represents the mesial surface of the teeth, a positive value on the y-axis represents the lingual surface of the teeth, and a positive direction on the z-axis represents towards the apex of the maxillary teeth.
