# Supplementary material for: Effective contribution ratio of the molar during sequential distalization using clear aligners and micro-implant anchorage: a finite element study
Source: Prog Orthod. 2023 Oct 9;24:35. doi: 10.1186/s40510-023-00485-0 (PMC10560653; doi:10.1186/s40510-023-00485-0)
Supplement: Supplementary file 2 — Additional file 2. Three-dimensional displacement values for the anterior teeth (mm). [file 40510_2023_485_MOESM2_ESM.docx]

**Supplementary file 2.** Three-dimensional displacement values for the anterior teeth (mm).

| Maxillary | Force magnitudes | Directions | x-axis | | | | | | y-axis | | | | | | z-axis | | | | | |
| --- | --- | --- | --- | --- | --- | --- | --- | --- | --- | --- | --- | --- | --- | --- | --- | --- | --- | --- | --- | --- |
|  |  | Groups | Set I | | | Set II | | | Set I | | | Set II | | | Set I | | | Set II | | |
|  |  | Models | A | B | C | A | B | C | A | B | C | A | B | C | A | B | C | A | B | C |
| Central incisor | 100g | Crown | 0.0014 | 0.0013 | 0.0014 | 0.0022 | 0.0021 | 0.0022 | -0.0729 | -0.0697 | -0.0707 | -0.0809 | -0.0776 | -0.0785 | 0.0164 | 0.0158 | 0.0174 | 0.0182 | 0.0175 | 0.0192 |
|  |  | Root | 0.0004 | 0.0005 | 0.0000 | 0.0004 | 0.0006 | 0.0000 | 0.0237 | 0.0228 | 0.0238 | 0.0264 | 0.0254 | 0.0265 | -0.0161 | -0.0145 | -0.0154 | -0.0180 | -0.0162 | -0.0172 |
|  | 150g | Crown | 0.0014 | 0.0013 | 0.0014 | 0.0022 | 0.0020 | 0.0022 | -0.0729 | -0.0680 | -0.0696 | -0.0809 | -0.0759 | -0.0774 | 0.0164 | 0.0155 | 0.0176 | 0.0182 | 0.0172 | 0.0193 |
|  |  | Root | 0.0004 | 0.0005 | -0.0002 | 0.0004 | 0.0006 | -0.0002 | 0.0237 | 0.0224 | 0.0237 | 0.0264 | 0.0249 | 0.0263 | -0.0161 | -0.0138 | -0.0149 | -0.0180 | -0.0156 | -0.0168 |
|  | 200g | Crown | 0.0014 | 0.0013 | 0.0014 | 0.0022 | 0.0020 | 0.0022 | -0.0729 | -0.0663 | -0.0685 | -0.0809 | -0.0742 | -0.0762 | 0.0164 | 0.0153 | 0.0178 | 0.0182 | 0.0169 | 0.0194 |
|  |  | Root | 0.0004 | 0.0005 | -0.0004 | 0.0004 | 0.0005 | -0.0004 | 0.0237 | 0.0219 | 0.0236 | 0.0264 | 0.0244 | 0.0261 | -0.0161 | -0.0132 | -0.0145 | -0.0180 | -0.0151 | -0.0163 |
| Lateral incisor | 100g | Crown | 0.0231 | 0.0219 | 0.0223 | 0.0263 | 0.0251 | 0.0254 | -0.0776 | -0.0765 | -0.0777 | -0.0850 | -0.0838 | -0.0850 | 0.0161 | 0.0172 | 0.0175 | -0.0850 | 0.0180 | 0.0183 |
|  |  | Root | -0.0035 | -0.0026 | -0.0023 | -0.0045 | -0.0034 | -0.0033 | 0.0248 | 0.0243 | 0.0242 | 0.0279 | 0.0274 | 0.0274 | -0.0154 | -0.0133 | -0.0137 | -0.0179 | -0.0158 | -0.0163 |
|  | 150g | Crown | 0.0231 | 0.0213 | 0.0220 | 0.0263 | 0.0245 | 0.0250 | -0.0776 | -0.0762 | -0.0778 | -0.0850 | -0.0835 | -0.0851 | 0.0161 | 0.0177 | 0.0181 | -0.0850 | 0.0185 | 0.0190 |
|  |  | Root | -0.0035 | -0.0022 | -0.0017 | -0.0045 | -0.0029 | -0.0027 | 0.0248 | 0.0241 | 0.0239 | 0.0279 | 0.0272 | 0.0271 | -0.0154 | -0.0124 | -0.0129 | -0.0179 | -0.0148 | -0.0155 |
|  | 200g | Crown | 0.0231 | 0.0206 | 0.0215 | 0.0263 | 0.0239 | 0.0245 | -0.0776 | -0.0760 | -0.0779 | -0.0850 | -0.0832 | -0.0852 | 0.0161 | 0.0183 | 0.0187 | -0.0850 | 0.0190 | 0.0197 |
|  |  | Root | -0.0035 | -0.0018 | -0.0011 | -0.0045 | -0.0024 | -0.0021 | 0.0248 | -0.0018 | 0.0235 | 0.0279 | 0.0271 | 0.0268 | -0.0154 | -0.0116 | -0.0121 | -0.0179 | -0.0138 | -0.0147 |
| Canine | 100g | Crown | 0.0612 | 0.0596 | 0.0598 | 0.0679 | 0.0661 | 0.0664 | -0.0425 | -0.0418 | -0.0415 | -0.0415 | -0.0410 | -0.0408 | 0.0167 | 0.0168 | 0.0173 | 0.0178 | 0.0176 | 0.0183 |
|  |  | Root | -0.0229 | -0.0223 | -0.0234 | -0.0269 | -0.0263 | -0.0274 | 0.0184 | 0.0186 | 0.0193 | 0.0182 | 0.0184 | 0.0191 | -0.0161 | -0.0152 | -0.0148 | -0.0176 | -0.0169 | -0.0164 |
|  | 150g | Crown | 0.0612 | 0.0590 | 0.0591 | 0.0679 | 0.0654 | 0.0656 | -0.0425 | -0.0413 | -0.0411 | -0.0415 | -0.0405 | -0.0403 | 0.0167 | 0.0171 | 0.0175 | 0.0178 | 0.0177 | 0.0185 |
|  |  | Root | -0.0229 | -0.0223 | -0.0237 | -0.0269 | -0.0262 | -0.0276 | 0.0184 | 0.0187 | 0.0199 | 0.0182 | 0.0184 | 0.0196 | -0.0161 | -0.0145 | -0.0142 | -0.0176 | -0.0164 | -0.0159 |
|  | 200g | Crown | 0.0612 | 0.0584 | 0.0583 | 0.0679 | 0.0647 | 0.0649 | -0.0425 | -0.0408 | -0.0406 | -0.0415 | -0.0399 | -0.0397 | 0.0167 | 0.0174 | 0.0178 | 0.0178 | 0.0179 | 0.0187 |
|  |  | Root | -0.0229 | -0.0223 | -0.0240 | -0.0269 | -0.0262 | -0.0278 | 0.0184 | 0.0188 | 0.0204 | 0.0182 | 0.0184 | 0.0201 | -0.0161 | -0.0139 | -0.0137 | -0.0176 | -0.0158 | -0.0153 |

The coordinate system was centered on each tooth( local coordinate system). A positive value on the x-axis represents the mesial surface of the teeth, a positive value on the y-axis represents the lingual surface of the teeth, and a positive direction on the z-axis represents towards the apex of the maxillary teeth.
